# Sentimental Analysis of COVID-19 Tweets Using Deep Learning Models

**DOI:** 10.3390/idr13020032

**Published:** 2021-04-01

**Authors:** Nalini Chintalapudi, Gopi Battineni, Francesco Amenta

**Affiliations:** 1 Telemedicine and Telepharmacy Centre, School of Medicinal and health products sciences, University of Camerino, 62032 Camerino, Italy; gopi.battineni@unicam.it (G.B.); francesco.amenta@unicam.it (F.A.); 2 Research Department, International Radio Medical Centre (C.I.R.M.), 00144 Rome, Italy

**Keywords:** COVID-19, sentimental analysis, word cloud, BERT, lockdown

## Abstract

The novel coronavirus disease (COVID-19) is an ongoing pandemic with large global attention. However, spreading false news on social media sites like Twitter is creating unnecessary anxiety towards this disease. The motto behind this study is to analyses tweets by Indian netizens during the COVID-19 lockdown. The data included tweets collected on the dates between 23 March 2020 and 15 July 2020 and the text has been labelled as fear, sad, anger, and joy. Data analysis was conducted by Bidirectional Encoder Representations from Transformers (BERT) model, which is a new deep-learning model for text analysis and performance and was compared with three other models such as logistic regression (LR), support vector machines (SVM), and long-short term memory (LSTM). Accuracy for every sentiment was separately calculated. The BERT model produced 89% accuracy and the other three models produced 75%, 74.75%, and 65%, respectively. Each sentiment classification has accuracy ranging from 75.88–87.33% with a median accuracy of 79.34%, which is a relatively considerable value in text mining algorithms. Our findings present the high prevalence of keywords and associated terms among Indian tweets during COVID-19. Further, this work clarifies public opinion on pandemics and lead public health authorities for a better society.

## 1. Introduction

The COVID-19 pandemic has prompted a sensational loss of human life worldwide and presents an extraordinary challenge to global health, food systems, and the work universe [1]. The monetary and social disturbance is demolishing by this pandemic. Many people are in danger of undernourishment, and it could increment by up to 132 million before the end of 2020 [2,3]. The dynamics of COVID-19, including the mortality, contagion factors, time of country virus, and initial deaths, were presented by the responsive differences between social media and financial markets from the after-effects of the severe virus spread [4].

The world that we knew up to this point has been changed and these days we live in a new situation in a never-ending transformation progress, in which how we live, relate, and speak with others has been modified for all time [5]. Inside this specific circumstance, virus risk is assuming a definitive job when educating, sending, and diverting the progression of data in the public arena. Coronavirus has represented a genuine pandemic risk and a broad challenge regarding control, readiness, reaction, and improvement by governments, wellbeing associations, stakeholders, and media broadcasting [4,5].

The present crisis due to COVID-19 is creating a socially advanced situation that is exceptional for health communities [6]. We are living in an exceptionally globalized world, where relocation adaptability, free travel among nations, and the turn of events and utilization of Information and Communication Technologies (ICTs) are essentially developed [7]. We should feature the developing interconnection between the world’s economies, which is reflected in the financial progress and individual knowledge [8].

Similarly, because of this virus outbreak, plenty of fake news and criticism on movements is continuously appearing on social media platforms. It disturbs communication among public health authorities and provokes high tension in the general public [9]. The study on social network analysis of novel virus sentimental analysis resulted in five relevant themes that range from positive to negative [10]. In this work, we considered the large dataset of tweets by Indian social media users. To do this, we defined a fine-tuned 12-layer Bidirectional Encoder Representations from Transformers (BERT) model, which is the latest deep-learning model for the analysis of textual data. The adoption of machine leering (ML) algorithms like logistic regression (LR), support vector machines (SVM), and single-layered LSTM (long-short term memory) models were done to compare the model performance with BERT. Based on this, this research work tries to answer the following research questions (RQ).

RQ1: What are the popular keywords that appeared in Indian tweets?

RQ2: How did these tweets affect public health systems?

RQ3: How do ML algorithms help to analyses people’s emotions or sentiments?

RQ4: How far can deep-learning 12-layer BERT model outperform the other three conventional ML models?

The rest of the paper was organized as follows: the materials and methods are presented in Section 2; experimental outcomes are presented in Section 3. Discussion and conclusions are explained in Section 4 and Section 5, respectively.

### COVID-19 Studies Related to Social Media Fake News

The increment in false propaganda via online sites is turning into a global issue. In spite of the fact that fake news is not vital, but it is currently troubling a result of online media prominence that provides cooperation and dissemination of novel ideas [11]. The situation of the COVID-19 epidemic shows the valuable effects of the origination of new data. The false data spreading can clearly impact individual mindset and change the viability of the countermeasures conveyed by governments [12].

The mis-information on social media can create panic and anxiety among COVID-19 patients, consequently alarms local governments and public authorities to urge citizens to confirm the genuineness of circulating stories [13]. Some studies presented about information accuracy and people’s behaviour in dealing with social media news associated with the COVID-19 pandemic [14,15,16]. The information diffusion of COVID-19 with big data analysis on major social media platforms presented an individual assessment of the address on a large scale to provide an exploration of epidemic rumours [17].

Twitter is a famous social media platform and microblogging medium where people post and share messages called “tweets”. About 500 million tweets per day and 200 billion tweets per year have appeared on Twitter since it has become an important data hotspot for web-based media conversation identified with public and global situations [18]. Unfortunately, it also a major source to create a global panic situation because of spreading fake news. Chakraborty K et al. 2020 described that most COVID-19 tweets are in positive sentiment, but users are frequently engaged in the spreading of negative tweets and not many useful words were found in the word frequency calculation of tweets [19].

In some words of Shadi S et al. 2020, the present pandemic creating rapidly spread rumours and conspiracy theories is like a virus that leads to real world dramatic values [20]. The authors of this work present machine learning approaches for automatic detection and identify narrative frameworks in support of such false propaganda. It is also studied that the effects of misleading the COVID-19 information and its political ideology that ultimately poisoned the public health [21].

The observations from the Nigerian study of the social media impact of COVID-19 pandemic reported platforms like Twitter, Facebook, and YouTube, which cannot be overemphasized with a plan of action for data dispersal. It indicates that these platforms have been manhandled as individuals cover-up under its secrecy to spread fake news and affect alarm among individuals from the overall population [22]. In high-population countries like India, it is even worse in spreading fake news online, which could be a potential threat to public health. On 24 March 2020, India declared a nationwide lockdown and alarmed public health experts to manage the challenges of COVID-19. However, these works do not address the accuracy and validation of tweets to a large extent.

In response, the study on social media false propaganda on COVID-19 during the lockdown time found the seven themes of fake news, including health, political, crime, entertainment, religious, religiopolitical, and miscellaneous, by analysing 125 Indian fake news [13] examples. There was work conducted by Bakur G et al. (2020) on sentiment analysis of Indian people during the national lockdown. The dataset used in this analysis contains very few tweets from 25 March 2020 to 28 March 2020. Results portrayed the positive strategy of the Indian government for imposing lockdown [23].

Huynh 2020 reported that plenty of rumours and fake stories are circulating in social media on COVID-19 and it is getting difficult to differentiate this false news from real news, but the accuracy or validity has not been questioned [24]. The validation and accuracy of such fakes on social media sites can help people, healthcare staff, and governments from unnecessary mental pressure. Such procedures give an overall clarification to in any case of unexceptional events, and fits conveniently into the perspective of the conspiracy theories. Therefore, to fill this research gap among literature on the tweet validation, we attempted deep-learning type machine-learning models, and also validate this model performance by comparing with another three conventional models.

## 2. Materials and Methods

### 2.1. Dataset

In this study, we incorporated the data of Indian user tweets from the Twitter website during the COVID-19 lockdown period in-country. The data set consisting of 3090 tweets were extracted from github.com (https://github.com/gabrielpreda/CoViD-19-tweets (accessed on 12 January 2021)) and it contains the cleaned tweets on topics such as COVID-19, coronavirus, lockdown, etc. The dataset consisting of extracted tweets from the Indian twitter platform was considered for analysis. The contained tweets focused on the topics of COVID-19, coronavirus, and lockdown, etc.

### 2.2. Data Analysis

**Figure 1:**
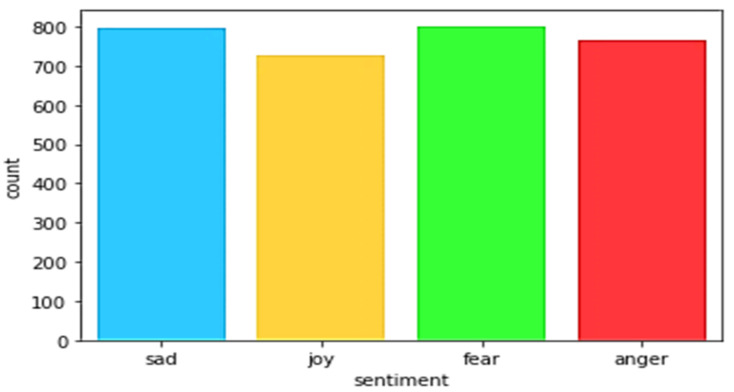
Tweets’ sentiment distribution.

Figure 1 presents the distribution of the tweets collected on the dates between 23 March 2020 and 15 July 2020, and the text has been labelled as fear, sad, anger, and joy. The given sample tweets are manually coded as four category sentiments by the investigators and each sentiment is mapped from 0 to 3 (fear:0, sad:1, anger:2, and joy:3). Examples of tweets presenting the sentiments had displayed in Table 1. The positive sentiments consisted of words “thank”, “well”, “great”, “agree”, “admit”, “good”, etc. and negative sentiments consisted of words “die”, “shit”, “trump”, “kill”, “spread”, “death”, etc.

We applied natural processing techniques (NLP), which are a form of ML that help tweet processing. Usually, NLP involves several text mining approaches, such as noise removal, by excluding stop and buzz words [25]. To enhance the BERT model performance measurements, immaterial substances, or text noises like accentuation marks, mathematical qualities, new-line characters were eliminated. Removing these elements moderates the size of the test space of conceivable capabilities and therefore improves the degree of execution.

**Figure 2:**
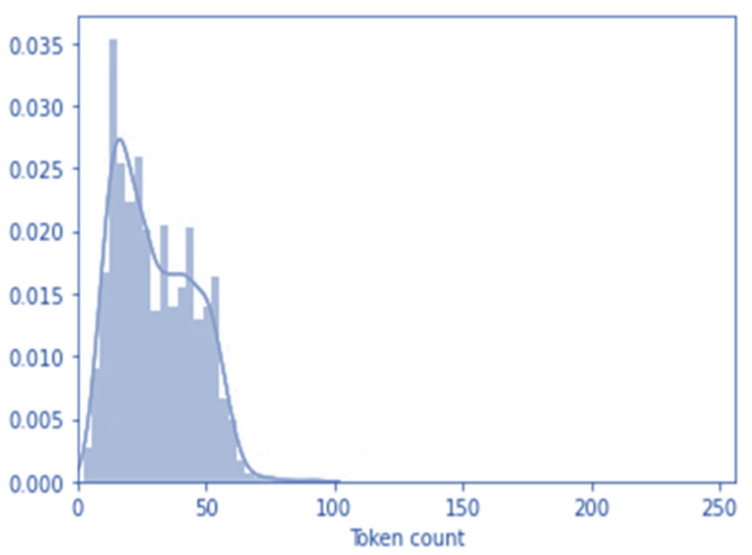
Tweets’ token length outcome.

As mentioned, to use pre-trained text weights in the BERT model, we have to configure the input features with encoding. This particular model works with fixed lengths, and we applied a maximum length of 100 tokens. The token length of all the tweets has been depicted in Figure 2. A high number of tweets is under 100 tokens.

### 2.3. Data Partitioning

To confirm, the classification model cannot be overfitting on the dataset. We divided the dataset into two portions such as training and testing. Trained tweets are helping to identify the data patterns, also reducing error rates, and testing the data set used for the assessment of model performance. Of the tweets, 85% were used for training purposes and 15% of tweets were used for testing purposes. The demographic information of tweets classification is presented in Table 2.

### 2.4. BERT Model

We implemented the BERT model for classifying the fake tweets. BERT is a new language presentation tool that stands for Bidirectional Encoder Representations from Transformers and was introduced in paper [26]. BERT has created a disturbance in the Machine Learning people group by introducing best in class, bringing about a wide assortment of NLP assessments. BERT’s key specialized advancement is applying the bidirectional preparing of Transformer, a mainstream consideration model, to language demonstrating. This is an advanced form, past advancements that took a look at text sequencing either from left to right or right to left and combined the left to the right model. The results from paper [26] explained that a language model that is bi-directionally prepared can have a more profound feeling of language setting and stream than single directional models.

#### 2.4.1. Model Training

In contrast to directional models that enable sequential reading of text input (right to left or left to right), the transformer encoder recognizes the total sequence of words at once. Thus, it is considered bidirectional, but it is a non-directional model with higher accuracy than other established models. This special characteristic allows the model to understand the text context. BERT has two models called the base model of 12 encoders and a large model of 24 encoders.

Model training in BERT has been completed by 15% tokens of input text language, which was randomly selected. These tokens further pre-processed by 80% are supplanted with a “MASK” token, 10% with an irregular word, and 10% utilize the first word. If we utilized “MASK” 100% of the time, the model would not delivers great symbolic portrayals for un-masked words. The un-masked tokens were as yet utilized for text setting, yet the model was upgraded for foreseeing masked words.

#### 2.4.2. Prediction of the Next Sentence

In BERT training, the model collects statement pairs as input and figures out how to anticipate if the second sentence in the pair is the resultant sentence in the original file. In training, half of the data sources are a pair where the subsequent sentence is the resulting sentence in the first archive, while in the other half an irregular sentence from the corpus is picked as the subsequent sentence. The supposition will be that the irregular sentence will be detached from the primary sentence.

The input source to BERT is the sum of the token, segment, and position embeddings. To assist the model to differentiate sentence pairs in training, input has processed as mentioned below.

⮚ A (CLS) token is embedded towards the initial statement and a (SEP) token is embedded toward the finish of each sentence.
⮚ A statement embedding demonstrates the Sentence A or Sentence B can be added to every token. Sentence embeddings are comparable in idea to token embeddings with a second statement vocabulary.
⮚ A positional inserting is added to every token to show its situation in the succession.

Word piece tokenization has been done by the BERT tokenizer: vocabulary displayed discrete characteristics of the language, and high-frequency vocabulary combinations repeatedly added. Keywords in Indian tweets were assessed by a word cloud diagram. Data was spitted into two subsets for training and validation purposes. Accuracy was further calculated to estimate the model accuracy.

### 2.5. Model Evaluation

To evaluate the efficiency of the BERT model, another three conventional models, such as LR, SVM, and LSTM, were further considered. LR is a kind of binary classification ML algorithm. It uses the weighted combination of the input text features and is authorized by the sigmoid function [27]. This function changes any real numerical input that ranges from 0 to 1. Instead of calculating the total number of words in a text document, we used T_f_-I_df_ (term frequency–inverse document frequency) for normalizing the words into a number. It estimates normalized count, such as the count of each word that is divided by a total number of documents, where the same word appears. We applied a similar ratio of dataset distribution (85:15) as followed in BERT modelling. SVM is another ML classification algorithm based on the identification of hyperplanes defined by data classes [28]. It operates on large data sizes and calculates the separation of data margin rather than matching key features. The similar approach of 85% of tweets is used for SVM model training.

Alternatively, we used the LSTM model, which is a type of recurrent neural network (RNN). LSTM network models are a sort of intermittent neural organization that can learn and recollect large information groups. They are expected for use with information that is contained in the long sequence of input data, up to 200 to 400-time steps [29]. That is why we considered LSTM as one of the accepted models for text analysis. The model can uphold various parallel information sequences and figures out how to remove features from data sequences. Like BERT, this model also trained by AdamW optimizer.

## 3. Results

**Figure 3:**
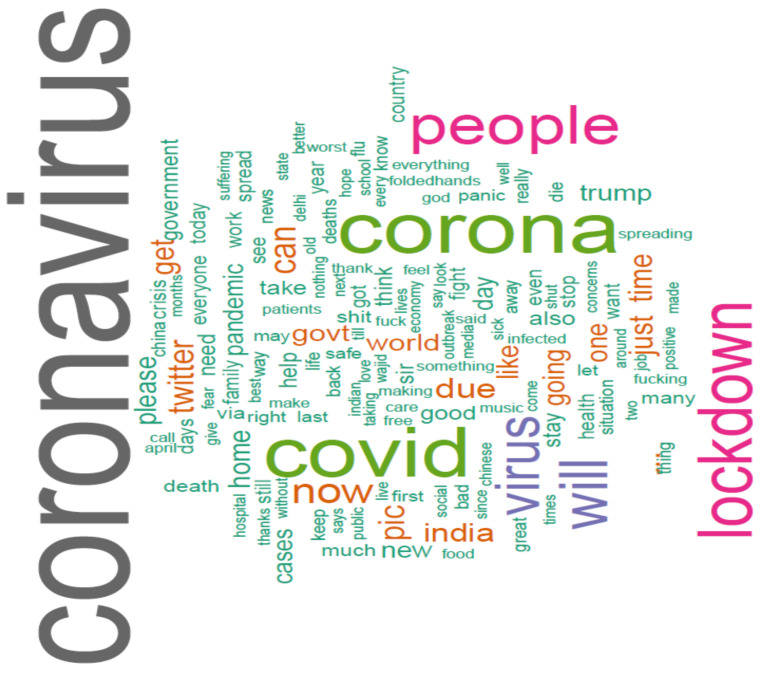
Word cloud of common words in Indian tweets.

**Figure 4:**
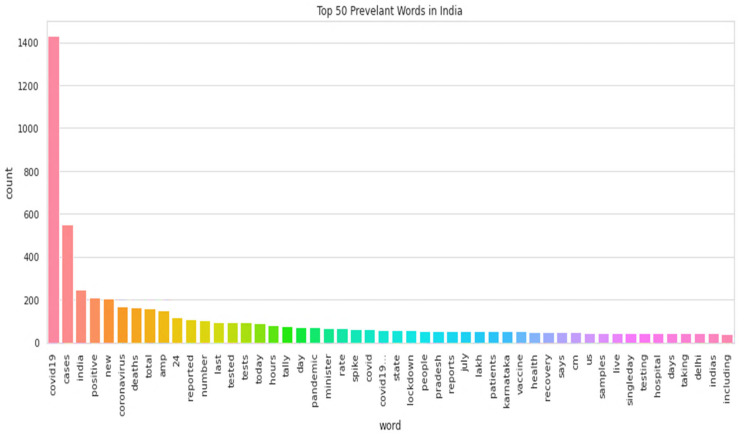
Top 50 keywords that appeared in Indian tweets.

The word cloud is a visual representation of words that usually appeared in texts. Figure 3 presents the word cloud of the Indian tweets’ dataset, and Figure 4 presents the top 50 keywords presented in Indian tweets.

For model performance evaluation, accuracy was considered the primary parameter. For fine-tuning and to duplicate the training features, we adopted the AdamW optimizer and applied the Adam algorithm by weight decay. Because of model training with the individual stage (or Epoch), training accuracy has resulted. The outcome test loss on a given test dataset has appeared as 0.594 and test accuracy appeared as 0.890, which presents that the model can generalize the tweets in a good manner. The model with 89% accuracy itself proved as a better solution in tweets classification. Table 3 presents the accuracy comparisons of four evacuated models.

The prediction accuracy of each sentiment tweet has been separately calculated by dividing sentimental data into training and validation sets. The mentioned tweets are fed as input for the model for training and the accuracy of each investigated sentiment has been assessed. The predicted accuracy values of each sentimental outcome can be observed in Table 4.

## 4. Discussion

The spread of pandemics causes vulnerability and anxiety among the global population. This sort of emergency, by not changing following explicit cut-off points, makes risk communication basic when planning to do better prevention strategies. If effective risk communication has been implemented, there is a chance of delivering messages to the public transparently and effectively. The key objective behind this is to diminish the knowledge gap between data investigators and recipients in understating public behaviour to fight against the pandemic. The fundamental components for risk reduction and anxiety assessment among the population are by taking rapid action from public health authorities and genuine data from governments.

Sentiment analysis majorly helps to understand people’s emotions in a particular event. Sentimental tweets about the pandemic have bound to be positive, suggesting that the public stayed confident despite a remarkable public wellbeing emergency. Keywords of positive sentiments normally communicated appreciation for community efforts and front-line workers to support vulnerable individuals of society, however, some keywords passed on negative opinions towards those who are at the frontline. A few words are attracted to joyful statements that can encourage doctors for fighting the war against COVID-19. Another type of positive estimation was the support of contamination measures to keep up general wellbeing guidelines like “stay safe”, “remain home” patterns.

India is the second-high pandemic that hit the country after the USA with 11.5 million infected cases and third position in deaths with a total of 160,427 [30]. The sentiment analysis of Indian tweets during COVID-19 lockdown obtained in this study have produced interesting results and presented the importance of further validation and experimental model advancement with more COVID-19 data, and supplementary methods. Models were subsequently created with extra information and strategies and utilizing BERT model tweet classification techniques would then be able to be utilized as self-governing techniques for autonomous classification of COVID-19 sentiment. The proposed model and results also similarly extended country-wise and global pandemic experiences in the future.

Recent trends in NLP suggest that text mining has increased its popularity in the advancement of big data analysis augmented computational competencies, and unstructured data analysis that enables the examination of huge linguistical datasets. The research presented in this paper with the use of the BERT sentiment analysis package in R produced a good exercise for evaluating the prediction accuracy of text outcomes. A similar analysis has been presented in [31] for the understanding of pandemic anxiety among Twitter users based on particular keywords. About 900,000 tweets are extracted from Twitter Application programming interface (API) and analysed using Naïve Bayes and logistic regression models. The model accuracy that appeared in short tweets is 91% and 74%, respectively. However, the main limitation of this study is all sentiments depend on the single word “fear” of USA citizens.

The study about precise ideas of netizens on the COVID-19 pandemic has identified twelve major topics that would emphasize sentiment topics and are highly related to health care problems [32]. Another interesting study on the mental health of Chinese youth for using Weibo messenger reported the most biased results. It highlighted negative sentiments in young minds during this outbreak [33]. In contrast, in this study, we highlighted four emotions of Indian netizens that associated both positive and negative sentimental categories. It is evident that from Table 3, the BERT model has outperformed the other three models in the classification of COVID-19 tweets. It produced 89% accuracy, which is much better than other models. Thus, it proves that the BERT model is the best solution for understanding the fake tweets while compared with other models. The present study has some limitations because it addresses only a single country of people’s emotions on social media sites on present pandemic, therefore, the generalization to global perspective on pandemics should be done in future studies.

## 5. Conclusions

In conclusion, our findings present the high prevalence of keywords and associated terms among Indian tweets during COVID-19. Based on the contents, the tweets are classified into four sentiments such as fear, sad, anger, and joy. Some words like “trump”, “kill”, “death”, “die” provoke people to have unnecessary fear and words such as “thank”, “well”, “good” create a positive setup in the healthcare authorities. These findings encourage local governments to impose fact-checkers on social media to overcome false propaganda. Previous literature only focuses on the effects of social media and its implications on the circulation of fake news but does not discuss the validation and classification of tweets. Thus, we applied a novel deep-learning model called BERT to achieve high classification accuracy in contrast to conventional ML models. Results provided enough evidence that the BERT model achieved 89% accuracy, which beats other models like LR, SVM, and LSTM. In this way, this work clarifies public opinion on pandemics and guides medical authorities, the public, and private workers to overcome needless anxiety during pandemics.

## Figures and Tables

**Table 1:** Sample tweets that express sadness, joy, fear, and anger sentiments on COVID-19.

Sad Sentiments	Joy Sentiments	Fear Sentiments	Anger Sentiments
# agree on the poor in India are treated badly their poor seek a living in Singapore and are treated like citizens they are given free medical treatment given food daily sim cards to call home to tell their family that they are fine if CoViD-19 case treated for in hospitals # I do not understand the point of demanding enough time before lockdown Italy has suffered because of half-hearted attempts and so is us now pm being the highest decision-making authority has to take the decision # UK records lowest daily virus death toll since the start of lockdown govt # because of CoViD 19 guidelines restricting visitation to hospitals and care centres john could not see his father in person before he died now, he is asking members of his community to donate I-pads that will be distributed to local medical centres	# good morning twittizens wish you a corona free day # finally accepts thanks to Modi media # how ted-ed is helping family’s students and teachers navigate the COVID-19 pandemic # the hardship of lockdown comes with the ease of blessing that Allah has brought family member together with my son asim town Punjab Pakistan # hoping that I would emerge on the other side of this healthier and more fit here’s my dinner salad # brother’s day 69 of lockdown from the archives of 2015 majnu ka tila (I’m normally lazy when it comes to archiving my photos and also the daily grind hinders the process but this lockdown boredom has given	# hackers use fake coronavirus maps to infect visitors with malware # I bet the idiot GB being is very concerned over the coronavirus not because of the health of their followers but because of the hit their bank account is going to take if meetings and assemblies are cancelled this is a worst-case scenario for them # when scares end # the thought of Jacob’s Nashville show getting cancelled makes my heart hurt soooooo bad please go away # is the making you afraid of what the future holds looking for a sense of peace call 877 why Islam you deserve to know	# maybe if I bolt my front door shut coronavirus will stay out # there are no injections for handle girls do not trust your bf # so, you no longer think that the coronavirus is a hoax perpetrated by the democrats exactly when was your epiphany moment # the Christian right and are the biggest threat to the world right now during this pandemic they outright evil their ignorance is death incarnate

**Table 2:** Demographic representation of tweets classification.

Sentiment	Label	Data Type	
Fear	0	Training	723
		Validation	128
Sad	1	Training	680
		Validation	115
Anger	2	Training	652
		Validation	115
Joy	3	Training	618
		Validation	109

**Table 3:** Accuracy comparisons of four models.

Model	Accuracy (in %)
Fine-tuned BERT	89%
LR	75%
SVM	74.75%
LSTM	65%

**Table 4:** Sentiment tweet distribution for model training.

Sentiment	Label	Data Type		Valid Tweets	Accuracy
Fear	0	Training	723	107	85.60%
		Validation	128		
Sad	1	Training	680	96	83.40%
		Validation	115		
Anger	2	Training	652	91	79.13%
		Validation	115		
Joy	3	Training	618	83	76.14%
		Validation	109		

## Data Availability

The authors would like to thank the data investigators Gabriel P and Arushi C. The tweets data and code can be available at https://github.com/gabrielpreda/CoViD-19-tweets (accessed on 12 January 2021).
